# Inequality in green space distribution and its association with preventable deaths across urban neighbourhoods in the UK, stratified by Index of Multiple Deprivation

**DOI:** 10.1136/jech-2024-222485

**Published:** 2024-11-12

**Authors:** Tran Thu Ngan, Ruoyu Wang, Christopher Tate, Mark Green, Richard Mitchell, Ruth F Hunter, Ciaran O'Neill

**Affiliations:** 1 Centre for Public Health, Queen's University Belfast, Belfast, UK; 2 Institute of Public Health and Wellbeing, University of Essex, Essex, UK; 3 Department of Geography & Planning, University of Liverpool, Liverpool, UK; 4 School of Health and Wellbeing, University of Glasgow, Glasgow, UK

**Keywords:** Health inequalities, DEATH, PREVENTION

## Abstract

**Background:**

This study investigated inequalities in the distribution of green space (GS) and the association between inequalities in amounts of GS and preventable deaths across urban neighbourhoods with different Index of Multiple Deprivation (IMD) scores in the UK.

**Methods:**

Data on preventable deaths, IMD, percentage of grassland and woodland, urban/rural, population size, and density were sourced for each of 6791 middle-layer super output areas (MSOAs) in England, 410 MSOAs in Wales, 1279 intermediate zones (IZs) in Scotland, and 890 super output areas (SOAs) in Northern Ireland (NI). While appreciating the potential for ecological fallacy we related area-based measures of deprivation to deaths. Concentration curves, Lorenz dominance tests, and negative binomial regression models were used to analyse the data.

**Results:**

In urban areas of England, Scotland, and NI, the percentage of grassland was significantly lower among the more deprived neighbourhoods (Lorenz test, p<0.0001). In England, a 1% increase in grassland area was associated with a 37% reduction in annual preventable deaths among the most deprived urban MSOAs (incidence rate ratio (IRR) 0.63, 95% CI 0.52 to 0.76). In NI and Scotland, a 1% increase in grassland area was associated with a 37% (IRR 0.63, 95% CI 0.43 to 0.91) and 41% (IRR 0.59, 95% CI 0.42 to 0.81) reduction in 5-year accumulated preventable deaths in the most deprived urban SOAs/IZs, respectively.

**Conclusions:**

Results suggest that investment in GS in urban areas may be an important public health prevention strategy. There is evidence that investments in the most deprived urban neighbourhoods where the highest inequality currently exists would see the largest effect on preventable deaths.

**WHAT IS ALREADY KNOWN ON THIS TOPIC**

**WHAT THIS STUDY ADDS**

- Except for Wales, there was significant inequality in the amount of GS across urban neighbourhoods with different Index of Multiple Deprivation (IMD) scores across the UK, to the advantage of those in more affluent areas.
- In urban areas of England, NI, and Scotland, a higher percentage of grassland was significantly associated with a lower number of preventable deaths among the most deprived group of IMD quintiles.

**HOW THIS STUDY MIGHT AFFECT RESEARCH, PRACTICE OR POLICY**

- Increased investment in GS in urban areas is urgently needed and should be prioritised in the most deprived neighbourhoods where provision is currently lowest and preventable deaths are highest.
- Investing in GS in the most deprived urban neighbourhoods is important for public health prevention strategies, particularly in light of the ongoing cost-of-living crisis and growing NHS issues in the UK.

## Background

 Investment in green space (GS) (green in the sense of being predominantly covered with vegetation^1^) in urban areas may help reduce preventable deaths, and generate various benefits that have been well-documented.^2^,^5^ Exposure to GS is associated with reduction of mortality and morbidity from chronic diseases, improvement of mental health and cognitive functioning, and reduction of obesity.^6^ These benefits might be realised through the provision of opportunities for physical activity/exercise and settings for relaxation and restoration from stress and fatigue; facilitating social interaction; and promoting social cohesion.^6 7^ GS also provides environmental benefits (eg, mitigate urban heat island effect, reduce air and noise pollution, function as biodiversity and nature conservation) and economic benefits (eg, reduced energy costs of cooling buildings and increase property values) which may benefit health outcomes.^8 9^

While investment in GS in urban areas may help reduce preventable deaths, where to prioritise investment is an important question for both equity and efficiency reasons. In European cities, accessibility to GS (within a 300 m linear distance of residences) tends to be higher within less deprived neighbourhoods than in neighbourhoods of lower socioeconomic position.^10^ This trend has been observed in Stockholm (Sweden),^11^ Porto (Portugal),^12^ Debrecen (Hungary),^13^ and in cities across the Netherlands.^14^ Similarly, a study conducted in England reported that local authorities with poorer GS provision had larger Black, Asian and minority ethnic populations and higher proportions of low-income households^15^—two population groups that already carry an excessively high burden of co-morbidities attributable to ill-health. UK data comparing the percentage of GS across urban neighbourhoods of different deprivation levels is absent. Further, research on the association between GS and preventable deaths among urban neighbourhoods of different deprivation levels is needed to identify geographic and socioeconomic priorities for interventions to improve health.

The aim of this study was to investigate: (1) the inequality in GS distribution in both urban and rural areas (for comparison); and (2) the association between availability of GS and preventable deaths across urban neighbourhoods in the UK, stratified by the Index of Multiple Deprivation (IMD).

## Methods

### Variables, measurements, and data sources

Statistical output geographies (from small to large) are: output areas (OAs), lower-layer super output areas (LSOAs), and middle-layer super output areas (MSOAs) for England and Wales; data zones (DZs) and intermediate zones (IZs) for Scotland; and small areas (SAs) and super output areas (SOAs) for Northern Ireland (NI) (see online supplemental table 1 in supplementary materials for details). Due to data availability, the primary geographic unit of analyses were the smallest geographic areas on which data on GS and preventable deaths were available. As such, it is MSOA for England and Wales, IZ for Scotland, and SOA for NI. There were 6791 MSOAs in England and 410 MSOAs in Wales; average MSOA population size is 7600 and 7200, respectively. There were 1279 IZs in Scotland with average population size ~4000, and 890 SOAs in NI with average population size ~2000. MSOA/IZ/SOA boundaries are mutually exclusive. Table 1 shows the sources of data.

**Table 1:** Variables, measurements, and data sources

Variables	Type	Data sources			
		England	Wales	Scotland	Northern Ireland
Preventable deaths	Number (integer)	2019 data of deaths by all causes (ICD-10) were sourced from ONS^18^ Preventable deaths were calculated using OECD 2022 definition^16^		2015–2019* data were provided by the Vital Events Team atNational Records of Scotland using OECD 2022 definition^16^	2016–2020* data were provided by Public Health Information and Research Branch (PHIRB), Department of Health using OECD 2022 definition^16^
Index of Multiple Deprivation	Ranking (rank first=most deprived) Quintiles	English Index of Multiple Deprivation (IMD) 2019^35^	Welsh Index of Multiple Deprivation (WIMD) 2019^36^	Scottish Index of Multiple Deprivation 2020 (SIMD) v2^37^	Northern Ireland Multiple Deprivation Measure (MDM) 2017^38^
Percentage of grassland or woodland	Proportion to total land area	UK Land Cover Map 2019 (20 m classified pixels imagery)^17^			
Percentage of green space areas	Proportion to total land area	Calculated by summing the areas of grassland and woodland (UK Land Cover Map 2019)^17^			
Population size	Number of people	Mid-2015 population estimatesMid-2017 population estimates (Scotland)			
Population density	Number of people per km^2^				
Settlement category	Categories (urban/rural)	Census 2011^18^			

* Due to the small number of deaths in these countries, the 5-year accumulated data were used. While time periods differ slightly, this is unlikely to affect results materially given the minor differences involved.

ICD-10International Classification of Diseases, 10th revisionOECDOrganisation for Economic Co-operation and DevelopmentONSOffice for National Statistics

### Outcome/dependent variable: preventable deaths

Preventable death refers to ‘causes of death that can be mainly avoided through effective public health and primary prevention interventions’ (that is, before the onset of diseases or injuries, to reduce incidence)^16^—for example, tuberculosis, ischaemic heart diseases, cerebrovascular diseases, cancer (depends on types), and chronic obstructive pulmonary disease.

Data on preventable deaths were provided by equivalent statistics offices in Scotland and NI (table 1) or calculated from data on deaths by all causes using the Organisation for Economic Co-operation and Development (OECD) lists of preventable and treatable causes of mortality (2022 version)^16^ in the cases of England and Wales.

### Independent variables

*Index of Multiple Deprivation(IMD)* is an overall relative measure of deprivation calculated by combining the weighted indices of deprivation from several domains (eg, income, employment, education, health, crime, access to services, and living environment). Each of these domains is based on a set of indicators. The number of domains and indicators are slightly different across each constituent country in the UK (see online supplemental table 2 for details).

IMD was originally measured and reported at LSOAs (England, Wales), DZs (Scotland), and SOAs (NI). As preventable deaths data are available at MSOAs for England/Wales, IZs for Scotland and SOAs for NI, the IMD was used as it is in the analysis for NI, while for the remaining countries IMDs were aggregated from the lower statistical output geographies. First, average rank and score for each MSOA/IZ were calculated by averaging all of the LSOA/DZ ranks and scores within each MSOA/IZ after these LSOA/DZ ranks and scores have been population weighted. These population weighted average ranks and average scores were then ranked, where the rank of one (most deprived) was given to the area with the ‘lowest average rank’ or ‘highest average score’. Due to data availability, rank of average rank and rank of average score were used in the analysis for England/Scotland and Wales, respectively.

*Percentage of grassland and percentage of woodland* were calculated by dividing the area of land covered by grass/wood by the total land area of a given geographic area. Data from the UK Centre for Ecology & Hydrology (UKCEH) Land Cover Map 2019 (20 m classified pixels imagery)^17^ were used for this calculation. In this dataset, there were 19 mutually exclusive classes of ‘land cover’ (see online supplemental table 3 for details). Grassland was made up of class 4–7 and 10 (improve grassland, neutral grassland, calcareous grassland, acid grassland, and heather grassland) while woodland comprised class 1 and 2 (deciduous woodland and coniferous woodland). Percentage of GS was calculated as the sum of percentage of grassland and woodland.

*Settlement category (urban/rural*) is a measure taken from Census 2011 data on the Office for National Statistics (ONS) Nomis website^18^ (see the ONS leaflet in online supplemental materials for details on how rural-urban classification was assigned to each MSOAs).

*Population and population density measures* are from mid-2015 (England, Wales, and NI) and mid-2017 (Scotland) population estimates which were also the data point used in the calculation of IMD.

*Median age* was also calculated for each MSOA/IZ/SOA using the above population estimates by age.

### Data analysis

Descriptive statistics (median and IQR) were used to describe the characteristics of the samples. Concentration curves and concentration index (C-index) were used to examine the inequality in the amount of GS among MSOAs/IZs/SOAs across the quintile of IMD. The concentration curve plots the cumulative percentage of GS areas (y-axis) against the cumulative percentage of the MSOA/IZ/SOA, ranked by IMD, beginning with the most deprived, and ending with the least deprived (x-axis). The line of equality is the 45° line, showing that irrespective of the geographic areas’ IMD, they have the same percentage of GS areas to total land areas. Lorenz (concentration) dominance tests were used to test if a concentration curve departs signiﬁcantly from an equal distribution.^19^ The concentration index (bounded between −1 and +1), defined as twice the area between the concentration curve and the line of equality, would be zero if there is no socioeconomic-related inequality.^19^ The negative/positive values indicate a distribution of GS that favours more/less deprived areas, respectively.

The factors that influenced preventable deaths were investigated using a negative binomial regression model, given the count nature of preventable deaths, and its distribution significantly differed from a Poisson distribution. In each country, the model was run only among the urban MSOAs/IZs/SOAs and stratified by each quintile of IMD to assess how associations varied by level of deprivation. The models have ‘count of preventable deaths’ as the outcome; ‘percentage of grassland/woodland’, ‘IMD’, ‘median age’, and ‘population density’ as independent variables; and ‘population size’ as an offset variable. The various statistical procedures were conducted in STATA version 15.0.

## Results

Characteristics of the data are presented in table 2. The median percentage of GS was highest in Wales (45%), followed by NI and England (24% and 21%, respectively), and then Scotland (16%). In all four countries, the median percentage of grassland/woodland/GS in urban areas was significantly lower than that of rural areas (Mann Whitney test, p<0.001).

**Table 2:** Characteristics of the data

	Rural	Urban	Total
**England, number of MSOAs (%)**	1192 (17.6)	5599 (82.4)	6791 (100.0)
	*Median (IQR*)	*Median (IQR*)	*Median (IQR*)
Number of preventable deaths	19 (12)	18 (12)*	18 (12)
Percentage of grassland†	33 (32)	12 (22)*	15 (26)
Percentage of woodland†	7 (9)	2 (8)*	3 (9)
Percentage of green space area†	43 (36)	17 (28)*	21 (33)
Population (mid-2015 population estimates)	7355 (2198)	7904 (2319)*	7834 (2343)
Population density (mid-2015) (per km^2^)	120 (164)	3271 (3494)*	2623 (3926)
Median age of the population (mid-2015)	46 (5)	38 (10)*	40 (10)
Percentage of population in age group 60–74‡	20 (4)	14 (6)*	15 (7)
**Wales, number of MSOAs(%)**	134 (32.7)	276 (67.3)	410 (100.0)
	*Median (IQR*)	*Median (IQR*)	*Median (IQR*)
Number of preventable deaths	21 (12)	21 (12)	21 (12)
Percentage of grassland†	66 (24)	27 (38)*	41 (46)
Percentage of woodland†	2 (8)	0* (1)*	0.02 (3)
Percentage of green space area	71 (23)	28 (43)*	45 (53)
Population (mid-2015 population estimates)	7120 (1960)	7413 (2405)*	7328 (2159)
Population density (mid-2015) (per km^2^)	98 (188)	1294 (2570)*	735 (2035)
Median age of the population (mid-2015)	46 (6.25)	40 (7)*	41.50 (7)
Percentage of population in age group 60–74‡	21 (5)	16 (4)*	17 (5)
**Scotland, number of IZs(%)**	288 (22.5)	991 (77.5)	1279 (100.0)
	*Median (IQR*)	*Median (IQR*)	*Median (IQR*)
Number of preventable deaths 2015–2019 (accumulated)	32 (17)	39 (28)*	38 (25)
Percentage of grassland†	28 (38)	4 (13)*	6 (22)
Percentage of woodland†	11 (19)	5 (12)*	7 (14)
Percentage of green space area†	45 (50)	11 (22)*	16 (34)
Population (mid-2017 population estimates)	3830 (1633)	4170 (1551)*	4101 (1645)
Population density (mid-2017) (per km^2^)	100 (0)	3200 (2200)*	27 (36)
Median age of the population (mid-2017)	46 (6)	41 (8)*	42 (9)
Percentage of population in age group 60–74‡	20 (5)	16 (5)*	17 (6)
**Northern Ireland, number of SOAs(%)**	340 (38.2)	550 (61.8)	890 (100.0)
	*Median (IQR*)	*Median (IQR*)	*Median (IQR*)
Number of preventable deaths 2016–2020 (accumulated)	13 (7)	15 (12)*	14 (10)
Percentage of grassland†	70 (38)	4 (20)*	17 (62)
Percentage of woodland†	7 (6)	0.3 (4)*	3 (7)
Percentage of green space area†	80 (30)	7 (25)*	24 (70)
Population (mid-2015 population estimates)	2291 (944)	1808 (707)*	1952 (870)
Population density (mid-2015) (per km^2^)	100 (100)	3000 (2900)*	13 (34)
Median age of the population (mid-2015)	29 (13)	30 (14)*	29 (13)

* Significant difference with p<0.001 (Mann-Whitney test between urban and rural).

† To total land areas.

‡ Data about percentage of population in age group 60–74 are not available for Northern Ireland.

IZsintermediate zonesMSOAsmiddle-layer super output areasSOAssuper output areas

**Figure 1:**
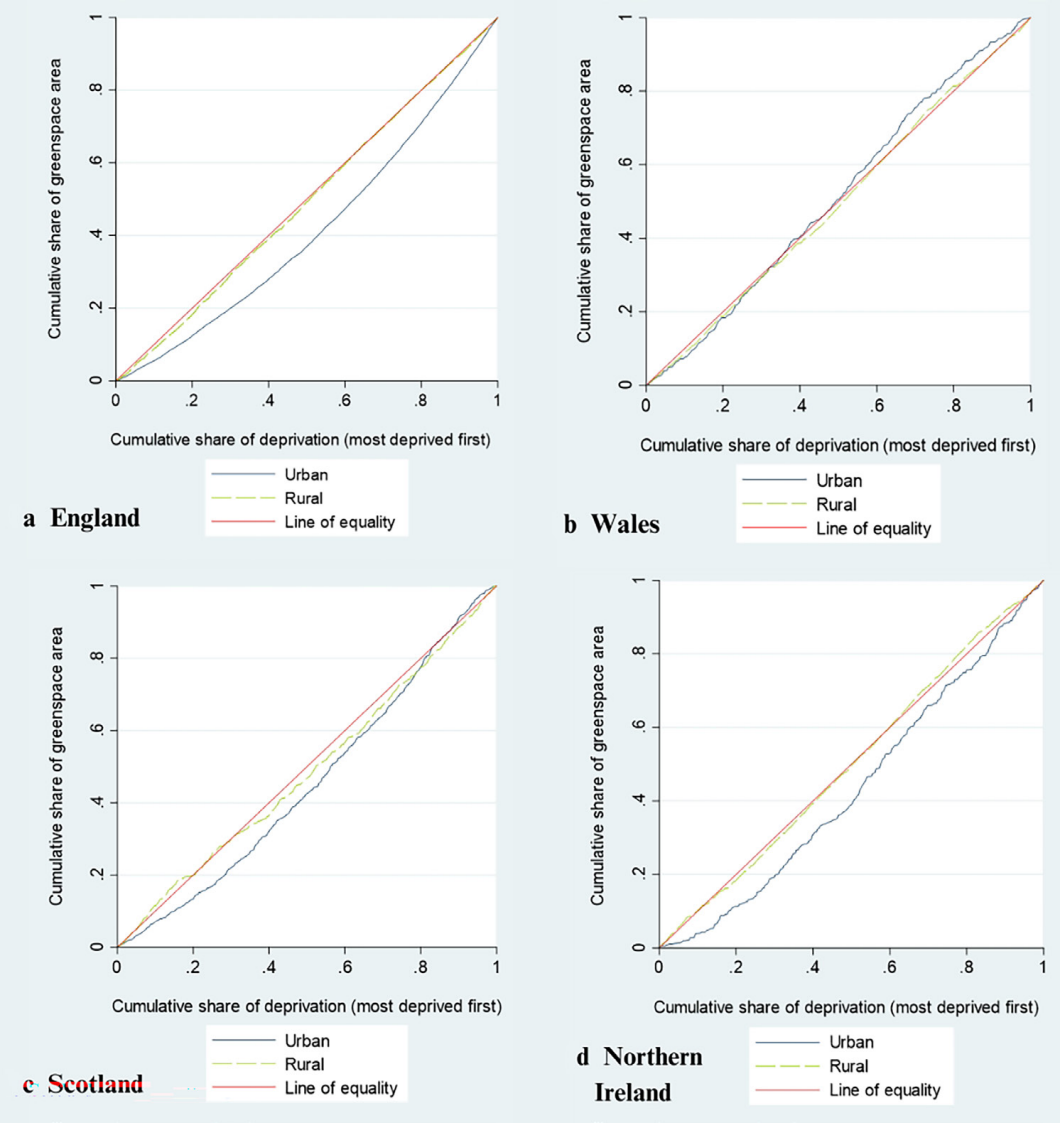
Concentration curves for percentage of green space areas to total land areas in England, Wales, Scotland, and Northern Ireland, by urban and rural areas.

Concentration curves for urban and rural MSOAs/IZs/SOAs of the four countries are plotted in figure 1a–d. In England, Scotland, and NI, the concentration curves of GS for urban areas depart signiﬁcantly from an equal distribution (Lorenz test, p<0.0001). These curves lie below the line of equality, indicating that the amount of GS is lower in more deprived areas. In contrast, the concentration curves of GS for rural areas do not depart significantly from the line of equality (Lorenz test, p>0.05), indicating that there is an equal amount of GS in rural areas of differing deprivation levels.

In Wales, both the concentration curves for urban and rural areas do not depart significantly from the line of equality (Lorenz test, p>0.05). As such, GS is distributed more equally across neighbourhoods with different levels of deprivation in both urban and rural areas of Wales.

The C-index and detailed results of the Lorenz dominance tests are presented in table 3. The magnitude of the index reflects the strength of the relationship between GS distribution and IMD as well as the degree of variability in the distribution. As such, the variability in distribution of GS among neighbourhoods with different IMD is largest in England (C-index=0.17), followed by NI (C-index=0.13) and Scotland (C-index=0.09).

**Table 3:** Concentration index of grassland, woodland, and green space in UK, by urban and rural

	Grassland*		Woodland*		Green space*	
	C-index (SE)	P value†	C-index (SE)	P value†	C-index (SE)	P value†
England						
Urban (n=5599)	0.17 (0.01)	<0.0001‡	0.16 (0.01)	<0.0001‡	0.17 (0.01)	<0.0001‡
Rural (n=1192)	0.002 (0.01)	0.81	0.06 (0.02)	0.0002‡	0.01 (0.01)	0.1093
Wales						
Urban (n=276)	−0.05 (0.03)	0.0703	0.26 (0.10)	0.0109‡	−0.02 (0.03)	0.3785
Rural (n=134)	−0.02 (0.01)	0.1711	0.33 (0.08)	0.0001‡	0.01 (0.01)	0.4617
Scotland						
Urban (n=991)	0.13 (0.03)	<0.0001‡	0.10 (0.02)	0.0151‡	0.09 (0.02)	<0.0001‡
Rural (n=288)	0.02 (0.03)	0.4681	0.10 (0.03)	0.1084	0.03 (0.02)	0.2072
Northern Ireland						
Urban (n=550)	0.12 (0.03)	0.0003‡	0.16 (0.05)	0.0008‡	0.13 (0.03)	<0.0001‡
Rural (n=340)	0.01 (0.01)	0.6389	−0.08 (0.02)	0.001‡	−0.003 (0.01)	0.7932

* Percentage of area to total land area (green space area=grassland area+woodland area).

† Results of the Lorenz (concentration) dominance tests (to test if a concentration curve departs signiﬁcantly from an equal distribution).

‡ Statistically significant at p<0.05.

C-indexconcentration index valueSEStandard error

Table 4 presents results of the negative binomial regression models investigating factors that influenced preventable deaths in urban areas, stratified by quintiles of IMD.

**Table 4:** Negative binomial regression model investigating factors that influenced preventable deaths in urban areas, stratified by quintiles of multiple deprivation

	Most deprivedIRR (95% CI)	SecondIRR (95% CI)	ThirdIRR (95% CI)	FourthIRR (95% CI)	Least deprivedIRR (95% CI)
England* (MSOA level)	**n=1335**	**n=1216**	**n=1082**	**n=915**	**n=1053**
Percentage of grassland	0.63 (0.52 to 0.76)†	0.81 (0.68 to 0.97)†	0.84 (0.70 to 1.02)	0.76 (0.63 to 0.93)†	0.84 (0.68 to 1.03)
Percentage of woodland	0.85 (0.60 to 1.20)	0.91 (0.66 to 1.26)	0.63 (0.43 to 0.91)†	0.71 (0.46 to 1.08)	0.56 (0.41 to 0.77)†
Median age of the population	1.05 (1.04 to 1.05)†	1.05 (1.04 to 1.05)†	1.05 (1.04 to 1.05)†	1.04 (1.04 to 1.05)†	1.05 (1.04 to 1.05)†
Population density (per 1000 m^2^)	0.95 (0.94 to 0.96)†	0.95 (0.95 to 0.96)†	0.95 (0.94 to 0.96)†	0.94 (0.93 to 0.96)†	0.95 (0.93 to 0.97)†
Wales* (MSOA level)	**n=50**	**n=38**	**n=55**	**n=63**	**n=70**
Percentage of grassland	1.15 (0.39 to 3.43)	1.98 (0.94 to 4.16)	0.96 (0.60 to 1.53)	0.91 (0.53 to 1.58)	0.74 (0.51 to 1.07)
Percentage of woodland	0.11 (0.00 to 169)	0.29 (0.01 to 16.6)	0.58 (0.16 to 2.18)	2.06 (0.74 to 5.72)	0.79 (0.38 to 1.63)
Median age of the population	1.06 (1.02 to 1.10)†	1.05 (1.03 to 1.07)†	1.04 (1.02 to 1.06)†	1.04 (1.02 to 1.07)†	1.03 (1.01 to 1.04)†
Population density (per 1000 m^2^)	1.00 (0.87 to 1.15)	1.08 (0.98 to 1.18)	0.97 (0.91 to 1.03)	1.02 (0.94 to 1.09)	0.97 (0.93 to 1.01)
**Scotland‡ (IZ level)**	**n=245**	**n=214**	**n=165**	**n=144**	**n=223**
Percentage of grassland	0.59 (0.42 to 0.81)†	0.77 (0.63 to 0.94)†	0.89 (0.71 to 1.10)	0.93 (0.72 to 1.20)	0.94 (0.72 to 1.21)
Percentage of woodland	1.20 (0.85 to 1.71)	0.84 (0.62 to 1.15)	0.90 (0.59 to 1.37)	0.73 (0.44 to 1.22)	0.99 (0.62 to 1.56)
Median age of the population	1.01 (1.00 to 1.02)†	1.01 (1.00 to 1.02)†	1.01 (1.00 to 1.01)†	1.02 (1.01 to 1.02)†	1.02 (1.01 to 1.03)†
Population density (per 1000 m^2^)	0.98 (0.97 to 0.998)†	0.98 (0.96 to 0.996)†	0.99 (0.98 to 1.01)	0.99 (0.97 to 1.01)	0.99 (0.97 to 1.01)
**Northern Ireland‡ (SOA level)**	**n=151**	**n=106**	**n=82**	**n=81**	**n=130**
Percentage of grassland	0.63 (0.43 to 0.91)†	1.35 (0.92 to 1.96)	0.97 (0.60 to 1.55)	1.34 (0.83 to 2.17)	0.75 (0.48 to 1.18)
Percentage of woodland	0.49 (0.11 to 2.26)	0.83 (0.35 to 1.96)	0.53 (0.15 to 1.87)	0.44 (0.11 to 1.62)	0.65 (0.24 to 1.71)
Median age of the population	1.02 (1.01 to 1.03)†	1.02 (1.01 to 1.03)†	1.02 (1.01 to 1.03)†	1.02 (1.01 to 1.03)†	1.02 (1.01 to 1.03)†
Population density (per 1000 m^2^)	1.01 (0.99 to 1.04)†	1.01 (0.98 to 1.04)	0.98 (0.94 to 1.02)	1.00 (0.95 to 1.04)	0.96 (0.91 to 1.01)

* Outcome variable is annual preventable deaths.

† P<0.05.

‡ Outcome variable is 5-year accumulated preventable deaths.

CIConfidence intervalIRRincidence rate ratioIZintermediate zones (average population size ~4000)MSOAmiddle layer super output areas (average population size ~7000)SOAsuper output areas (average population size ~2000)

In urban areas of England, Scotland, and NI, higher percentage of grassland was significantly associated with a lower number of preventable deaths among the most deprived group of IMD quintiles. In England, with every 1% increase of grassland area in an MSOA, the incidence rate ratio (IRR) suggests that annual preventable deaths count in that MSOAs were lower by 37% (IRR 0.63, 95% CI 0.52 to 0.76). In NI and Scotland, with every 1% increase of grassland area in an SOA/IZ, the IRRs suggested that 5-year accumulated preventable deaths count in that SOA/IZ were lower by approximately 37% (IRR 0.63, 95% CI 0.43 to 0.91) and 41% (IRR 0.59, 95% CI 0.42 to 0.81), respectively.

In Wales, the percentage of grassland was not significantly associated with the number of preventable deaths. There were also no significant associations found between woodland area and the number of preventable deaths in Wales, Scotland, and NI. In England, the associations were observed among the third and least deprived group of IMD quintiles.

## Discussion

This study reported two key findings. First, except for Wales, there was significant inequality in the amount of GS among urban neighbourhoods with different IMD across the UK, to the advantage of those in more affluent areas. Second, in urban areas of England, NI, and Scotland, a higher percentage of grassland was significantly associated with a lower number of preventable deaths among the most deprived group of IMD quintiles.

### Inequality in the amount of GS in urban areas

The amount of GS was lowest among the most deprived areas of England, Scotland, and NI. This result echoes a previous 2008 study in England which reported that those with greater exposure to GS were more likely to be less deprived (r^2^=−0.28, p<0.0001).^20^ This trend is also similar in Australian and European cities. For example, GS accounted for 20% of land area in the most affluent areas compared with 12% among the most low-income neighbourhoods of Adelaide (Australia).^21^ In Porto (Portugal), the number of accessible GS was smallest in the most deprived neighbourhoods and the distance to GS increased with neighbourhood deprivation.^12^ In the Netherlands, lower presence and quality of GS were observed in neighbourhoods with a low socioeconomic status compared with those with a high socioeconomic status.^14^ In Germany, neighbourhoods with high multiple deprivation index scores, including low average income, low levels of educational attainment and high unemployment rates, tended to have access to smaller areas of GS than their more affluent counterparts.^22 23^ With the known health benefits of GS, this discrepancy may help to explain the wide health inequalities in urban areas in which the poorest and most vulnerable are most impacted.

The unequal distribution of GS in urban areas demonstrates the need to target interventions at more deprived urban areas. Studies have shown that GS brings about greater benefit to those of lower socioeconomic position than those who belong to the more privileged groups, particularly in mental health and social integration.^24^,^26^ This suggests that GS distribution mirrors the ‘inverse care law’,^27^ in that it is least available to the communities who need it most, and for whom it might have greatest impact. Knowledge of where the greatest need for investment in GS exists can, therefore, help increase the return on investment, and cost-effectiveness, of such interventions.^5^ In addition, community involvement in the development and management of GS should be carried out as it can increase the sense of ownership, and potentially encourage use of the facilities.^528^,^30^

### GS and preventable deaths in urban areas

The analysis found a statistically significant association between the percentage of grassland and number of preventable deaths among the most deprived urban areas in three of the four countries of the UK. It is perhaps notable that the exception, Wales, was where inequalities in the amount of GS were not significant (in either urban or rural areas). We observed that a 1% increase in grassland area was significantly associated with a 37–41% reduction in the number of preventable deaths. However, these associations were not consistent across other quintiles of multiple deprivation. This finding adds to the evidence from Ruoyu *et al* (NI)^31^ and Mitchell and Popham^20^ (England) which reported that among the least green areas, incidence rates for all-cause mortality of the most income deprived quartile was 1.93 (95% CI 1.86 to 2.01) times higher than that of the least deprived.^20^ These findings suggest that a greater impact can be achieved from investment in GS targeting the most deprived urban areas. It also echoes the fact that preventable deaths are massively socially patterned, and therefore influenced by socioeconomic factors rather than just a lack of healthcare intervention.

Although the study results show the association between lower number of preventable deaths and higher percentage of GS area, investment should not solely focus on increasing the existence of GS but also on the accessibility and quality of such GS.^5 30 32^ In reality, the land area covered by GS is often bigger than the area of GS that is accessible to the public. A European Environment Agency study from 38 countries reported that area of publicly available GS only accounted for 3% of the total city area (though GS of all kinds made up 42% of the total land areas).^33^ Further research investigating the link between health outcomes and accessibility to GS should be explored.

### Strength and limitations

To the best of our knowledge, this is the first UK-wide study that used data at the small area level to look at inequalities in the amount of GS among neighbourhoods and the associations between levels of GS in urban areas and preventable deaths across levels of deprivation. Data on different parameters were sourced from various government public databases or directly from the national statistic agencies before linking with each other in order to create a robust dataset for secondary analysis.

There were several limitations to the present study. First, we relate area-based measures of deprivation to deaths assuming implicitly that those living and dying in deprived areas are themselves more likely to be deprived. This may not be the case—being rich in a deprived area may be positively correlated with avoidable death—however, in the absence of data on access, preventable death and deprivation at the individual level, we are obliged to proceed recognising the potential for ecological fallacies. We also aggregated IMD to larger statistical output geographies (from LSOA/DZ to MSOA/IZ) for which it was not designed; thus, it might introduce ecological fallacies into our analyses. The ecological nature of our analyses mean that we cannot rule out residual confounding (eg, greener areas may just pick up neighbourhood deprivation indirectly). Besides, the use of statistical output geographies as a unit of analysis carries an inherent risk of error with respect to individuals, especially those living on the boundary of the MSOAs/IZs/SOAs as very nearby GS in the adjacent unit may be more relevant (for actual use) than GS at the other side of their own administrative unit.

Second, the study used ‘land cover’ data which show the visible surface of land (eg, crops, grass, broad-leaved forest or built-up area). ‘Land use’ data might be a better measurement for analysis since they indicate how people are using land (eg, agriculture, forestry, recreation, or residential use) which is part of the mechanism on how GS may benefit health. Data showing frequency of access to GS, activities performed, and length of each visit could have improved on these measures, although such national level data are rarely available to researchers. As such data are not available, we had to resort to ‘land cover’ data under the assumption that land cover and use are positively correlated with each other.

Thirdly, the negative binomial models could be adjusted further for quality of GS (using, for example, the normalised difference vegetation index) which as an indicator may influence the use and activity within GS and, in turn, the number of preventable deaths. Such data are, again, not commonly available to researchers and represent an important research gap. It may be reasonable to assume that the most deprived neighbourhoods not only experience a lower quantity, but also a lower quality of GS compared with less deprived areas.^32^ It is plausible that the impact on preventable deaths may be even greater than the current estimates if targeted investment was directed towards increasing the area of GS as well as the quality.

Apart from the quality of GS, we appreciate a range of potential confounding variables could have been added to our analysis. These might include economic indicators, indicators of healthcare provision, crime rates and pollution, and perhaps include lagged values of these. However, many of these would be expected to be correlated with the IMD (which was calculated by combining the weighted indices of deprivation from several domains including income, employment, education, health, crime, access to services, and living environment). Each of these domains is based on a set of indicators and issues of endogeneity may arise in estimation.

Finally, this is a secondary analysis of cross-sectional data so the interpretation of the results should be treated with caution. We cannot conclude that the relationship between GS and preventable deaths is causal. It is conceivable that GS and preventable deaths are related through their relationships with other unobserved variables. Future research should consider extending our work using longitudinal data, including study designs that can identify cause and effect.^34^ Work examining the return on investments in GS could further examine this.

## Conclusions

Inequality in the amount of GS in urban areas exists across three of the four countries of the UK, with the tendency that the most deprived urban areas have the least GS provision. The strongest association between GS area and the number of preventable deaths was observed in the most urban deprived neighbourhoods. These results support the argument for increased investment in GS, and that this should be prioritised in the most deprived neighbourhoods where provision is currently lowest and preventable deaths are highest. Investing in GS in the most deprived neighbourhoods, therefore, is important for prevention, particularly in light of the ongoing cost-of-living crisis and growing NHS issues in the UK.

## supplementary material

10.1136/jech-2024-222485 online supplemental file 1

10.1136/jech-2024-222485 online supplemental file 2

## Data Availability

All data relevant to the study are included in the article or uploaded as supplementary information.
