# Supplementary material for: Inequality in green space distribution and its association with preventable deaths across urban neighbourhoods in the UK, stratified by Index of Multiple Deprivation
Source: J Epidemiol Community Health. 2024 Nov 12;79(2):e222485. doi: 10.1136/jech-2024-222485 (PMC11874280; doi:10.1136/jech-2024-222485)
Supplement: online supplemental file 1 [file jech-79-2-s001.pdf]

Government Statistical Service

The 2011 Rural-Urban Classification for Output Areas in England

- In 2011 in England 43.7 million people (82.4% of the population) lived in **urban areas (settlements of more than 10,000 people)**.
- 9.3 million people lived in **rural areas** (17.6% of the population), i.e. in smaller towns (less than 10,000 people), villages, hamlets or isolated dwellings.
- Rural areas make up 85% of the land area.
- It is important to distinguish between rural and urban areas when analysing social and economic statistics as the populations and businesses can differ in their make-up (for example rural areas tend to have higher proportions of older people).
- The **opportunities**, **challenges** and **barriers** for **businesses**, the **services** people receive and their **quality of life** can also differ markedly between rural areas and larger towns and cities.
- Wherever possible the **Rural-Urban Classification** should be used for statistical analysis.
- At its most detailed the Rural-Urban Classification assigns areas to one of **six rural or four urban settlement/context types**.
- The classification has been applied to a range of geographic datasets to facilitate analysis, including Census geographies and postcodes. It can be used in aggregated form to classify and analyse larger geographic areas to suit the level at which data are available. For example, a **Rural-Urban Classification for Local Authority Districts**.
- This leaflet explains the basics of the Classification, its use, and where to find more information.

If printed (use short edge print setting) this can be folded along the dotted lines into three to form a leaflet

- **Urban areas are** the connected **built up areas** identified by Ordnance Survey mapping **that have resident populations above 10,000 people** (2011 Census)**.**
- **Rural areas are those areas that are not urban**, i.e. consisting of settlements below 10,000 people or are open countryside.
- For the 2011 Census, England was divided into 171,372 **Output Areas** (OAs) which on average have a resident population of 309 people. OAs are the smallest geographic unit for which Census data are available. Their geographical size will vary depending on the population density.
- **Each OA is assigned as urban or rural** based on whether its (population-weighted) centre is within or outside a built up area of greater than 10,000 people. The rural and urban labels can then be used to analyse Census or other data, to allow comparisons between rural and urban areas.
- A simple rural / urban statistical split may not be sufficient to reflect the diversity of rural and urban areas.
- By looking at dwelling densities for every 100m x 100m square and the density in squares at varying distances around each square, and then comparing these ‘density profiles’ for different types of settlement, the settlement form across the country can be mapped and this allows **every OA to be assigned a settlement type**. For the first time this has been done to provide a typology of urban areas (previously only classed as ‘urban’).
- As well as settlement form, the wider **context of each settlement** can be determined by looking at dwelling densities at greater distances, up to 30km beyond, to identify sparsely populated areas and thus **settlements in a sparse setting**.
- The **Rural-Urban Classification of Output Areas** (shown below) consists of six rural and four urban settlement/context combinations.

The Rural-Urban Classification for Output Areas

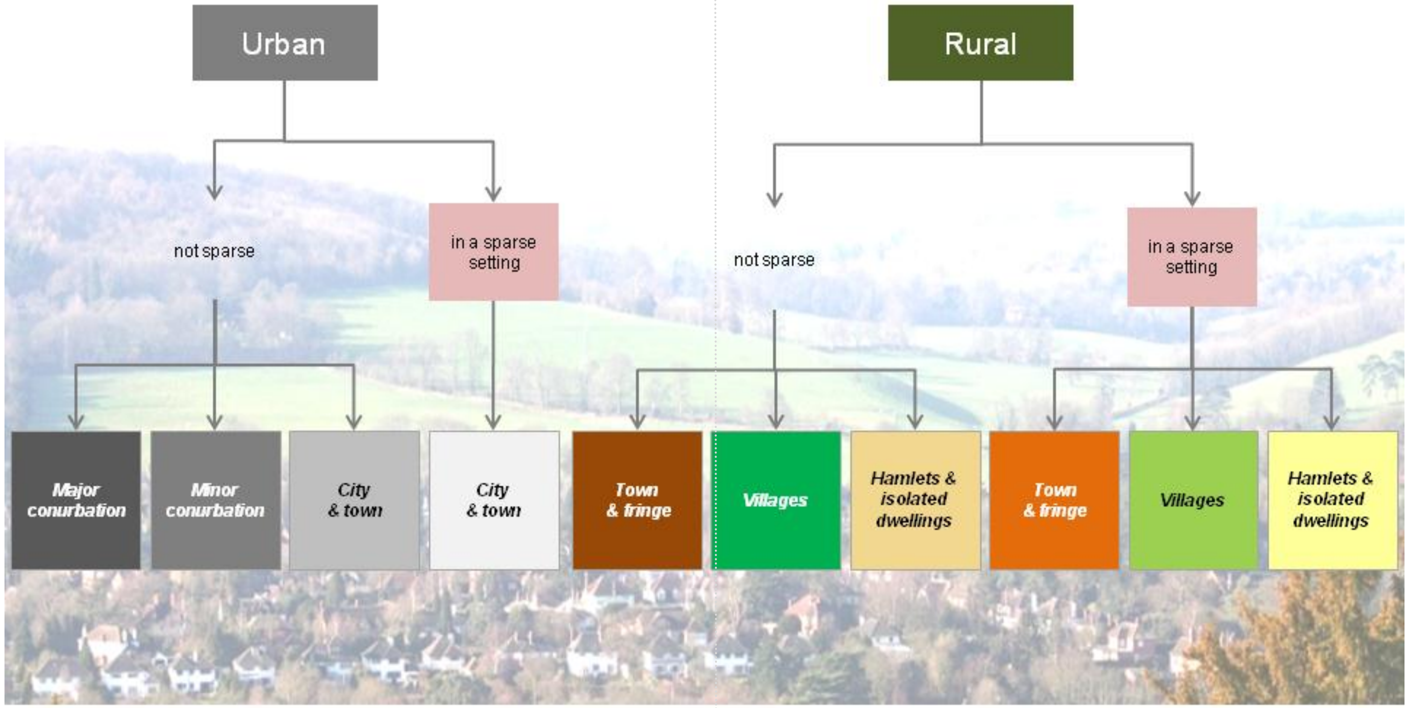

The 2011 Rural-Urban Classification for Output Areas in England

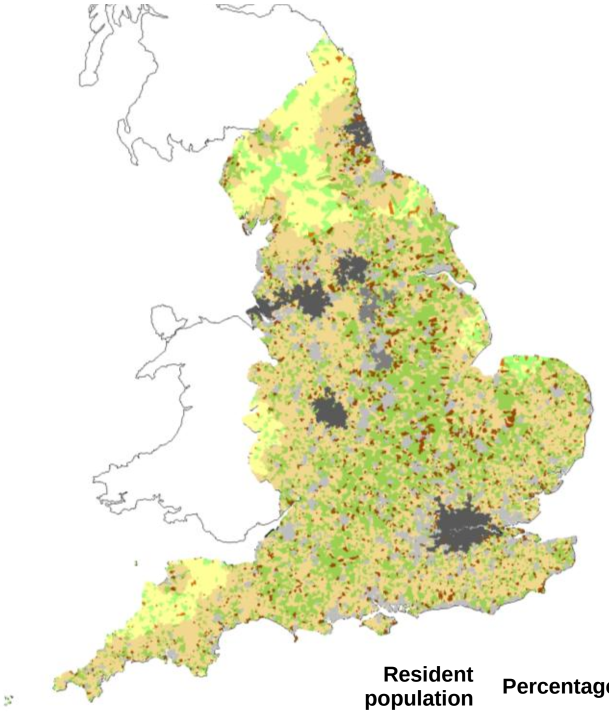

	Resident population	Percentage
Urban major conurbation	18,783,700	*35.4*
Urban minor conurbation	1,906,100	*3.6*
Urban city and town	22,889,800	*43.2*
Urban city & town *in a sparse setting*	88,900	*0.2*
**Total Urban**	**43,668,600**	***82.4***
Rural town and fringe	4,470,700	*8.4*
Rural village	2,772,700	*5.2*
Rural hamlet & isolated dwellings	1,619,000	*3.1*
Rural town & fringe *in a sparse setting*	186,300	*0.4*
Rural village *in a sparse setting*	157,700	*0.3*
Rural hamlet & isolated dwellings *in a sparse setting*	137,400	*0.3*
**Total Rural**	**9,343,900**	***17.6***
**Total England**	**53,012,500**	***100.0***

Source: 2011 Census, Rural-Urban Classification

- ❑ The OA classification has been aggregated to classify other small area geographies, i.e. **Lower-Layer Super OAs** (populations of 1,000 to 3,000), **Middle-Layer Super OAs** (populations of 5,000 to 15,000), and **Wards** (average population 6,000), although some of the settlement types are combined at these levels.

The Rural-Urban Classification for Higher Level Geographies

- ❑ OA-level information can be aggregated to suit data at larger spatial scales, including **Local Authority Districts** (LADs; see *Useful Links* for a separate leaflet).
- ❑ LADs are classified based on the share of their population that lives in **rural** or **'rural-related'** areas (i.e. hub towns), as shown in the table below.
- ❑ **Hub towns** are built-up areas (defined by Ordnance Survey) with a population of **10,000 to 30,000** that meet specific criteria relating to **dwelling and business densities**, suggesting the potential to **serve the wider rural hinterland**.

Category	Description	Broader category
Urban with Major Conurbation	Less than 26% living in rural settlements and hub towns	Predominantly Urban
Urban with Minor Conurbation	Less than 26% living in rural settlements and hub towns	
Urban with City and Town	Less than 26% living in rural settlements and hub towns	
Urban with Significant Rural	At least 26% but less than 50% living in rural settlements and hub towns	Urban with Significant Rural
Largely Rural	At least 50% but less than 80% living in rural settlements and hub towns	Predominantly Rural
Mainly Rural	At least 80% living in rural settlements and hub towns	

Warning: Output areas may cover a large area of open countryside and yet be still urban if most of the population lives in an urban settlement. Rural is a matter of settlement form and dwelling density rather than the economic function or the character or use of the land.

Most local authorities classed as rural will include urban populations and vice versa.

The Classification is very unlikely to be useful for planning purposes, and should be fully understood before using for any purpose beyond statistical analysis.

Useful links

- **Rural-Urban Classifications for**
  - **Output Areas (OA)**
  - **Lower Layer Super Output Areas (LSOA)**
  - **Middle Layer Super Output Areas (MSOA)**
  - **Wards**
  - **Postcodes (ONS Postcode Directory)**

  are available via the Office for National Statistics Open Geography Portal (under *Download Products*) as are built-up area boundaries https://geoportal.statistics.gov.uk/geoportal/catalog/main/home.page
- **2011 Rural-Urban Classification for Local Authority Districts** (user guide, methodology, lookup table, list of hub towns and leaflet) https://www.gov.uk/government/statistics/2011-rural-urban-classification-of-local-authority-and-other-higher-level-geographies-for-statistical-purposes or via 'Documents' on the Open Geography Portal above.
- **Office for National Statistics 2011 Census data** (available on NOMIS with the Rural-Urban Classification) https://www.nomisweb.co.uk
- **A guide to Rural-Urban Census analysis on NOMIS** https://ww.gov.uk/government/publications/2011-census-rural-analysis-a-guide-to-nomis
- **Key statistics for built up areas** https://www.ons.gov.uk/ons/rel/census/2011-census/key-statistics-for-built-up-areas-in-england-and-wales/index.html
- **Statistical Digest of Rural England** https://www.gov.uk/government/organisations/department-for-environment-food-rural-affairs/series/statistical-digest-of-rural-england
- **Rural statistics for local authorities and Local Enterprise Partnerships (LEPs)** https://www.gov.uk/government/statistical-data-sets/rural-statistics-local-level-data-sets
- **Welsh statistics** https://www.wales.gov.uk/topics/statistics/?lang=en

The Rural-Urban Classification is a Government Statistical Service product developed by the Office for National Statistics; the Department for Environment, Food and Rural Affairs; the Department for Communities and Local Government; and the Welsh Assembly Government, in collaboration with Sheffield and Nottingham Universities.

Enquiries
census.customerservices@ons.gsi.gov.uk
rural.statistics@defra.gsi.gov.uk

Produced by Defra Rural Statistics, May 2015
Map © Crown Copyright and database rights 2013; Ordnance Survey Licence No. 100022861
