# Supplementary material for: Inequality in green space distribution and its association with preventable deaths across urban neighbourhoods in the UK, stratified by Index of Multiple Deprivation
Source: J Epidemiol Community Health. 2024 Nov 12;79(2):e222485. doi: 10.1136/jech-2024-222485 (PMC11874280; doi:10.1136/jech-2024-222485)
Supplement: online supplemental file 2 [file jech-79-2-s002.pdf]

Journal of Epidemiology and Community Health

Supplementary materials for article

Inequality in green space distribution and its association with preventable deaths across urban neighbourhoods in the United Kingdom, stratified by index of multiple deprivation

Tran Thu Ngan[1], Ruoyu Wang[1 2], Mark A. Green[3], Rich Mitchell[4], Christopher Tate[1], Ruth F. Hunter[1], Ciaran O'Neill[1]

[1] *Centre for Public Health, Queen's University Belfast, Belfast, United Kingdom*

[2] *Institute of Public Health and Wellbeing, University of Essex, Essex, United Kingdom*

[3] *Department of Geography & Planning, University of Liverpool, Liverpool, United Kingdom*[4] *School of Health & Wellbeing, University of Glasgow, Glasgow, United Kingdom*

* Correspondence to:
Tran Thu Ngan, Ph.D.,
Centre for Public Health, School of Medicine, Dentistry and Biomedical Sciences
Queen's University Belfast, Belfast, BT12 6BA, United Kingdom
Email: n.t.tran@qub.ac.uk
ORCID: 0000-0003-2771-9878

Table of contents

Table S1. Statistical output geographies in the four countries of the United Kingdom (Census 2011)
Table S2. Index of multiple deprivation (IMD) in the United Kingdom
Table S3. UKCEH Land Cover Classes

Table S1. Statistical output geographies in the four countries of the United Kingdom (Census 2011)

	**Statistical output geographies (small to large)**		
**England and Wales**	**Output Areas (OAs)**<br><br>Resident population: 100-625 persons<br><br>England & Wales: 181,408 OAs	**Lower layer Super Output Areas (LSOAs)**<br>Resident population: 1,000-3,000 persons<br>Usually made up of 4-5 OAs<br>England: 32,844 LSOAs<br>Wales: 1,909 LSOAs	**Middle layer Super Output Areas (MSOAs)**<br>Resident population: 5,000-15,000<br>Usually made up of 4-5 LSOAs<br>England: 6,791 MSOAs<br>Wales: 410 MSOAs
**Scotland**	**Data Zones (DZ)**<br>Resident population: 500-1000<br>Scotland: 6,976 DZs	**Intermediate Zones (IZs)**<br>Resident population: 2,500-6,000<br>Scotland: 1,279 DZs	
**Northern Ireland (NI)***	**Small Areas (SAs)**<br>Resident population: 400 (range: 98-3,072)<br>NI: 4,537 SAs	**Super Output Areas (SOAs)**<br>Resident population: 2000<br>NI: 890 SOAs	

** For Census 2021, NI has new statistical output geographies: Data Zones and Super Data Zones. These replaced 2011 Census Output Areas.*

References:

1. Office of National Statistics (ONS). 2011 Census geographies. Available at: https://www.ons.gov.uk/methodology/geography/ukgeographies/censusgeographies/2011censusgeographies
2. Scottish Borders Council. Scottish Borders Datazones and Intermediate Zones. Available at: https://www.scotborders.gov.uk/council-2/research-data/5
3. Northern Ireland Statistics and Research Agency (NISRA). Output geography for Census 2011. Available at: https://www.nisra.gov.uk/support/geography/output-geography-census-2011

Table S2. Index of multiple deprivation (IMD) in the United Kingdom

The index of multiple deprivation (IMD) is an overall relative measure of deprivation calculated by combining the weighted indices of deprivation from several domains. Each of these domains is based on a set of indicators (please see references for the full list of indicators in each country of the UK).

	England	Wales	Scotland	Northern Ireland
**Statistical unit**	Lower layer Super Output Areas	Lower layer Super Output Areas	Data zones	Super output areas
**Domains of deprivation and Domain weight (%)**	1. Income Deprivation (22.5%)	1. Income (22%)	1. Income (28%)	1. Income (25%)
	2. Employment Deprivation (22.5%)	2. Employment (22%)	2. Employment (28%)	2. Employment (25%)
	3. Education, Skills and Training Deprivation (13.5%)	3. Education (14%)	3. Education (14%)	3. Education, Skills and Training (15%)
	4. Health Deprivation and Disability (13.5%)	4. Health (15%)	4. Health (14%)	4. Health and disability (15%
	5. Crime (9.3%)	5. Community safety (5%)	5. Crime (5%)	5. Crime and disorder (5%)
	6. Barriers to Housing and Services (9.3%)	6. Access to services (10%)	6. Access to services (9%)	6. Access to services (10%)
		7. Housing (7%)	7. Housing (2%)	
	7. Living Environment Deprivation (9.3%)	8. Physical environment (5%)		7. Living environment (5%)

References:

1. Ministry of Housing, Communities & Local Government. The English Indices of Deprivation 2019 – Research report. Available at: https://www.gov.uk/government/publications/english-indices-of-deprivation-2019-research-report
2. Statistics for Wales, Welsh Government. Welsh Index of Multiple Deprivation (WIMD) 2019 - Technical report. Available at: https://www.gov.wales/sites/default/files/statistics-and-research/2023-10/welsh-index-multiple-deprivation-2019-technical-report.pdf
3. Scottish Government. Scottish Index of Multiple Deprivation (SMID) 2020 Technical Notes. Available at: https://www.gov.scot/publications/simd-2020-technical-notes/
4. Northern Ireland Statistics & Research Agency (NISRA). Northern Ireland Multiple Deprivation Measures 2017. Journal of Statistical and Social Inquiry Society of Ireland Vol. XLVIII. Available at: https://www.tara.tcd.ie/handle/2262/91639

Table S3. UKCEH Land Cover Classes

UKCEH Land Cover Class	LC identifier	UKCEH Land Cover Class	LC identifier	UKCEH Land Cover Class	LC identifier
**Deciduous woodland**	**1**	Fen	8	Supralittoral rock	15
**Coniferous woodland**	**2**	Heather	9	Supralittoral sediment	16
Arable	3	**Heather grassland**	**10**	Littoral rock	17
**Improve grassland**	**4**	Bog	11	Littoral sediment	18
**Neutral grassland**	**5**	Inland rock	12	Saltmarsh	19
**Calcareous grassland**	**6**	Saltwater	13	Urban*	20
Acid grassland	7	Freshwater	14	Suburban*	21

** Not mutually exclusive with other classes (1-19)*

Definition for each class is described in the reference (page 19-24).

In our study, grassland category was made up of class 4-7, and 10 while woodland category was made up of class 1 and 2.

Reference:

1. UK Centre for Ecology & Hydrology (UKCEH). User guide for UKCEH Land Cover Maps 2017, 2018 and 2019. Available at: https://www.ceh.ac.uk/sites/default/files/2021-11/lcm2017-2019product_documentation_v1_5_1.pdf
